# Stress and Corticosteroids Modulate Muscarinic Long Term Potentiation (mLTP) in the Hippocampus

**DOI:** 10.3389/fncel.2017.00299

**Published:** 2017-09-21

**Authors:** Efrat Shavit Stein, Ze’Ev Itsekson Hayosh, Andreas Vlachos, Nicola Maggio

**Affiliations:** ^1^Department of Neurology, The Chaim Sheba Medical Center at Tel Hashomer Ramat Gan, Israel; ^2^Department of Neuroanatomy, Institute of Anatomy and Cell Biology, Faculty of Medicine, University of Freiburg Freiburg, Germany; ^3^Department of Neurology and Neurosurgery, Sackler School of Medicine, Tel Aviv University Tel Aviv, Israel; ^4^Sagol School of Neuroscience, Tel Aviv University Tel Aviv, Israel; ^5^Talpiot Medical Leadership Program, The Chaim Sheba Medical Center at Tel Hashomer Ramat Gan, Israel

**Keywords:** stress, corticosteroids, muscarinic LTP, cholinergic transmission, Alzheimer’s disease, hippocampus

## Abstract

Stress influences synaptic plasticity, learning and memory in a steroid hormone receptor dependent manner. Based on these findings it has been proposed that stress could be a major risk factor for the development of cognitive decline and dementia. Interestingly, evidence has been provided that stress also affects muscarinic, i.e., acetylcholine (ACh)-mediated neurotransmission. To learn more about the impact of stress and steroids on synaptic plasticity, in this study, we investigated the effects of stress on muscarinic long term potentiation (mLTP). We report that multiple, unpredictable exposure to stress depresses carbachol (0.5 μM)-induced mLTP, while this effect of stress is not observed in hippocampal slices prepared from mice exposed only to a single stressful procedure. Furthermore, we demonstrate that activation of distinct steroid hormone receptors is involved in stress-mediated alterations of mLTP. Activation of mineralocorticoid receptors (MR) promotes mLTP, while glucocorticoid receptor (GR) activity impairs mLTP. These effects of multiple unpredictable stress on mLTP are long-lasting since they are detected even two weeks after the last stressful experience. Thus, multiple unpredictable events rather than a single stressful experience affect mLTP in a steroid hormone receptor dependent manner, suggesting that chronic unpredictable stress can lead to lasting alterations in hippocampal cholinergic plasticity.

## Introduction

Stress has a major impact on synaptic plasticity, learning and memory (Joëls et al., 2008; Joëls and Baram, 2009). Exposure to repeated, unpredictable stress is a risk factor for neurological deficits in neurodegenerative diseases (Goldstein, 2011). Specifically, chronic stress has been shown to accelerate the onset and progression of cognitive decline in Alzheimer’s disease (AD) patients (Simard et al., 2009; Solas et al., 2010; Dhikav and Anand, 2011). The mechanisms underlying this phenomenon are not completely understood, although evidence has been provided that stress may increase hippocampal neurodegeneration (Jeong et al., 2006) via tau hyperphosphorylation (Sotiropoulos et al., 2011) and A-beta pathology (Srivareerat et al., 2009).

Exposure of animals to various forms of stress has major effects on the ability of hippocampal neurons to express electrically induced (tetanic) long term potentiation (LTP) of excitatory synapses (Pavlides et al., 2002; Alfarez et al., 2003; Karst and Joëls, 2003; Maggio and Segal, 2007, 2011, 2012b; Krugers et al., 2010; Segev et al., 2014; Sharvit et al., 2015). Indeed, robust experimental evidence exists that corticosteroids mediate the effects of stress on synaptic plasticity (Pavlides et al., 1994, 1995, 1996; Krugers et al., 2005; Wiegert et al., 2005; Maggio and Segal, 2007, 2009a,b, 2012a; Joëls et al., 2009). Interestingly, stress has been also linked to alterations in acetylcholine (ACh)-mediated neurotransmission (Kaufer et al., 1998; Farchi et al., 2007), e.g., by mediating alternative splicing of the primary ACh-hydrolyzing enzyme ACh-esterase (Farchi et al., 2007). Considering: (1) the relevance of the ACh in cognitive functions (Levey, 1996; Hut and Van der Zee, 2011); (2) the fact that deficits in cholinergic neurotransmission have been reported in a number of neuropsychiatric diseases, including AD (Yan and Feng, 2004; Berson et al., 2012); and (3) our earlier work on corticosteroid receptor mediated modulation of tetanus-induced LTP (Maggio and Segal, 2007, 2009b, 2012b), it appeared well warranted to test whether stress interferes with ACh-mediated neural plasticity in a corticosteroid dependent manner. Specifically, we were interested in testing whether this effect of stress is seen under conditions of repeated, unpredictable stress rather than after a single stressful procedure only.

In this study, the mechanisms involved in stress-induced modulation of muscarinic long term potentiation (mLTP) were assessed. mLTP is a form of synaptic plasticity that is induced by exposing hippocampal slices to carbachol, a muscarinic ACh-analog (Markram and Segal, 1990; Auerbach and Segal, 1996). Indeed, multiple, unpredictable exposure of stress depresses hippocampal mLTP in a corticosteroid-receptor dependent manner. This effect is long-lasting, since it can be detected 2 weeks after exposure to stress. Thus, our findings reveal a new mechanism on how stress affects synaptic plasticity via modulation of ACh-mediated synaptic plasticity, which may be important in the context of stress-related cognitive decline and the development of dementia.

## Materials and Methods

### Mice and Stress Protocol

The study was approved by the Sheba Medical Center Institutional Animal Care and Use Committee (protocol number 698/11) which adheres to the national law and NIH rules. Mice were maintained in a 12-h light/dark cycle with food and water available ad libitum. Three months old male C57BL/6 mice were exposed either to acute stress or to multiple stress between 9:00 AM and 11:00 AM. The multiple stress protocol consisted of a repeated, unpredictable stressful procedure including a 15-min forced swim stress in a bucket of water (FSS) on the first day, a 30 min stay on an elevated platform stress (EPS) on the second day and a 2 h restraint in a narrow tube restrain stress (RS) on the third day. Age- and time-matched animals were exposed to a single stressful procedure only, which either was FSS, EP or RS. Control animals were kept in different cages and similarly handled, however not exposed to any stressful procedure. Experimental assessment of mLTP was carried out 1 day after the exposure to the stressful procedure.

### Pharmacology and Drugs

The mineralocorticoid receptors (MR) agonist aldosterone (100 μg mg/kg; Sigma) and the glucocorticoid receptor (GR) agonist dexamethasone (100 μg/kg; Sigma) were injected intraperitoneally (*i.p*.) 1.5 h prior to slice preparation. The MR antagonist spironolactone (Sigma) and the GR antagonist RU38486 (mifepristone, Sigma) were injected *i.p*. at a concentration of 20 mg/kg, 1 h prior to stress exposure. Control animals were *i.p*. injected with vehicle only. Drugs were prepared according to the manufacturer instructions and stored at −20°C (Maggio and Segal, 2012c; Maggio et al., 2017). For the *in vitro* experiments, drugs were superfused into the recording medium at given concentrations with special care to prevent changes in temperature, pH, flow rate, or degree of oxygenation of the artificial cerebrospinal fluid (ACSF).

### Slice Procedures and Electrophysiology

Extracellular recordings in acute slices prepared from dorsal hippocampus were performed as previously described (Maggio and Segal, 2007, 2012a). Following anesthesia with ketamine/xylazine (100 mg/kg and 10 mg/kg, respectively), animals were rapidly decapitated and 400 μm hippocampal slices were prepared using a vibroslicer (NVSLM1 vibroslice, World Precision Instruments, Serasota, FL, USA). Slices were incubated for 1.5 h in a humidified, carbogenated (5% CO_2_ and 95% O_2_) gas atmosphere at 33 ± 1°C and were perfused with ACSF [containing (in mM) 124 NaCl, 2 KCl, 26 NaHCO_3_, 1.24 KH_2_PO_4_, 2.5 CaCl_2_, 2 MgSO_4_, and 10 glucose, pH 7.4] in a standard interface chamber. Recordings were made with a glass pipette containing 0.75 M NaCl (4 MOhm) placed in the stratum radiatum of CA1 as described previously (Maggio and Segal, 2007). Input-output curves were acquired from each slice prior to experimental assessment. Responses were digitized at 5 kHz and stored on a computer. Spike 2 software (Cambridge Electronic Design, Milton, Cambridge, England) was used for data acquisition. Data are reported as means ± SEM. Where appropriate, statistical analysis was performed with analysis of variance (ANOVA) followed by *post hoc* Tukey’s comparisons.

## Results

### Exposure to Stress Affects mLTP at Schaffer Collateral-CA1 Synapses

**Figure 1:**
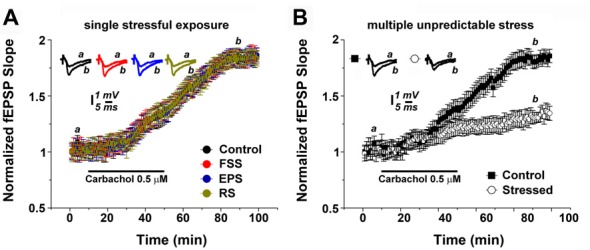
Multiple unpredictable stressful events modulate muscarinic long term potentiation (mLTP).** (A)** Exposure of animals to a single stressful procedure does not affect mLTP. **(B)** mLTP is depressed in hippocampal slices of animals which underwent multiple unpredictable stress exposure. Averaged field excitatory postsynaptic potentials (fEPSP) are plotted vs. time. Representative traces at indicated times (a,b) are shown on top of each section, *n* = 12 slices for each experiment, refer to text for statistics. FSS, Forced swim stress; EPS, Elevated platform test; RS, Restrain stress.

Three months old male C57BL/6 mice were exposed to the following stress protocols: (1) a multiple, unpredictable stressful procedure including a FSS on the first day, an EPS (EP) on the second day and RS on the third day; or (2) a single stressful procedure of either FSS, EP or RS (see, Figure 1). Age- and time-matched control animals were not exposed to any stressful procedure but otherwise handled similarly.

Hippocampal slices were prepared the day after the (last) exposure of stress. In order to test for the effects of the two stress protocols on mLTP of Schaffer collateral-CA1 synapses, 0.5 μM carbachol was bath applied for 40 min after acquiring a 10-min baseline of evoked field excitatory postsynaptic potentials (fEPSP). In slices of non-stressed control animals, carbachol reliably induced a slow increase in fEPSP slope (= mLTP), which continued to increase after the washout of carbachol, reaching its maximal value at about 80 min (1.79 ± 0.06). The plateau level was maintained till the end of the recordings (Figure 1A).

In hippocampal slices prepared from animals 1 day after a single stressful procedure, i.e., either after FSS, EP or RS, mLTP was not affected in comparison to controls: both the dynamics and magnitude of mLTP were indistinguishable between the groups (Figure 1A; *n* = 12 slices per each group; one-way ANOVA; *p* = 0.67, *F* = 0.50, *post hoc* Tukey’s; Figure 1A).

However, exposing animals to the multiple stress protocol had a major effect on the ability to express mLTP (Figure 1B). Specifically, at 80 min the fEPSP slope values were 1.29 ± 0.07 for the stressed animals compared to 1.81 ± 0.07 for the control animals (*n* = 12 slices per each group; *p* < 0.001; Figure 1B). We conclude from these experiments that multiple stressful experiences, rather than a single stressful event, alter cholinergic plasticity at Schaffer collateral-CA1 synapses.

### Corticosteroid Receptor Activation Modulates mLTP

**Figure 2:**
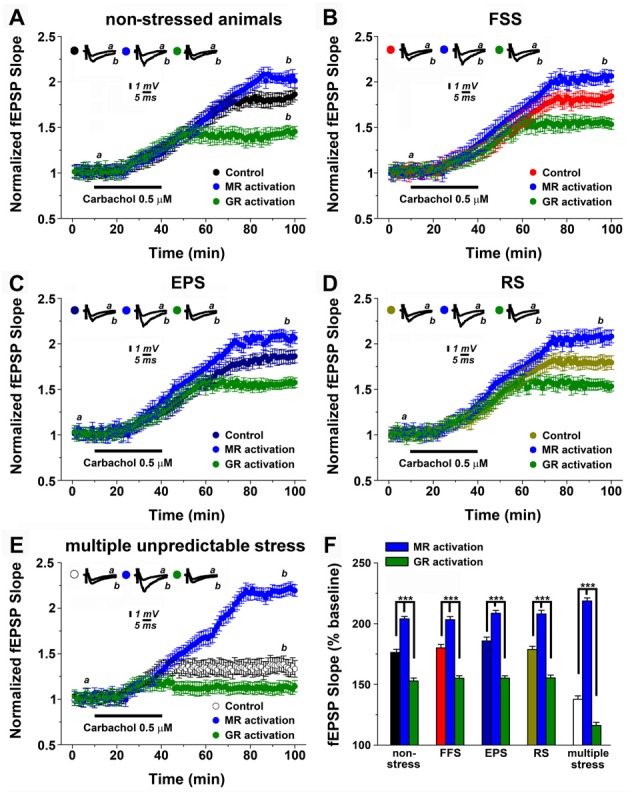
Mineralocorticoid and glucocorticoid receptor (GR) mineralocorticoid receptors (MR, GR) modulation differentially affect mLTP after stress. **(A)** In non-stressed animals, MR and GR activation (respectively, single *i.p*. injection of the MR agonist aldosterone, 100 μg/kg, i.e., MR activation or single *i.p*. injection of the GR agonist dexamethasone 100 μg/kg, i.e., GR activation) enhances or depresses mLTP, respectively. **(B–D)** A similar effect of MR and GR is observed upon a single stressful exposure. In these experiments, MR activation was obtained by blocking GR with mifepristone (20 mg/kg) prior to stress exposure, while GR activation was obtained by blocking MR with spironolactone (20 mg/kg) prior to stress exposure. **(E)** MR and GR inhibition results in stronger effects on either enhancement or depression of mLTP, with GR inhibition preventing alterations in mLTP following multiple unpredictable stress. **(F)** Summary graph and combined analysis of experimental data. Further details of the results and the statistical comparisons are described in the text. Statistical analyses were made with two-ways analysis of variance (ANOVA), followed by *post hoc* Tukey’s comparisons. FSS, Forced swim stress; EPS, Elevated platform test; RS, Restrain stress.

It is well-established that steroid hormone activation differentially modulates the ability of neurons to express electrically induced (tetanic) LTP of excitatory neurotransmission (McEwen, 1999; Joëls and Krugers, 2007; Joëls et al., 2008; Segal et al., 2010; Maggio and Segal, 2012b). Therefore, in a first step, we tested whether activation of MR or GR modulates mLTP-expression in non-stressed control animals (Figure 2A). GR or MR activation were achieved by single *i.p*. injections of aldosterone (aldo, 100 μg/kg, an agonist of MR, i.e., MR activation) or dexamethasone (dexa, 100 μg/kg, an agonist of GR, i.e., GR activation) 1.5 h prior to slice preparations.

Figure 2A shows that MR or GR activation have distinct effects on the ability of neurons to express mLTP: while MR activation enhanced mLTP (2.02 ± 0.049 at 95 min of recordings compared to 1.80 ± 0.072 of controls), GR activation impaired mLTP (1.43 ± 0.064; *p* < 0.0001).

### Effects of Corticosteroid Receptor Activation on mLTP after Acute Stress

We then tested for the role of MR and GR after exposure to a single stressful event. Previous work disclosed that blocking GR with mifepristone (20 mg/kg) under conditions of stress has similar effects to MR activation in non-stressed animals, while spironolactone (20 mg/kg) a blocker of MR results in GR activation under stress conditions (Avital et al., 2006). Drugs were injected 1 h prior to the stressful experience, i.e., FSS (Figure 2B), EPS (Figure 2C) or RS (Figure 2D).

Indeed, GR-inhibition under stress conditions, i.e., MR activation improved mLTP, while MR inhibition under stress conditions, i.e., GR activation had the opposite effect (see, Figures 2A–D). The differential effects of steroid hormone activation did not differ from those occurring in the non-stressed animals (two-way ANOVA for multiple comparisons among means, *F* = 128.4, *p* < 0.001, Figure 2F). These experiments confirm and extend the role of MR and GR activation on mLTP modulation and provide additional support for the notion that a single stressful exposure to stress does not modulate the ability of neurons to express hippocampal cholinergic plasticity.

### Inhibition of GR Prevents Alterations in mLTP Under Conditions of Multiple, Unpredictable Stress

What is the effect of corticosteroid receptors under conditions of multiple, unpredictable stress? In this set of experiments, animals were injected with either mifepristone or spironolactone 1 h prior each stressful experience (Figure 2E). Similar to the results described above, under stress conditions GR-inhibition, i.e., MR activation improved mLTP, while MR-inhibition, that is GR activation had a detrimental effect on the ability of neurons to express mLTP (*F* = 226.2, *p* < 0.001). However, GR-inhibition in fact restored mLTP back to levels seen under non-stressed conditions, or in animals that received only one stressful procedure (Figure 2E). In contrast MR-inhibition almost completely blocked the ability of neurons to express mLTP (*F*_interaction between factors_ = 25.5, *p* < 0.001; Figure 2F). We conclude from these results that multiple stressful exposures to stress impair the ability of neurons to express mLTP while activation of MR and GR has a more profound effect on mLTP with GR-inhibition restoring the ability of neurons to express mLTP.

### *In Vitro* Effects of Corticosteroid Receptor Activation on mLTP

**Figure 3:**
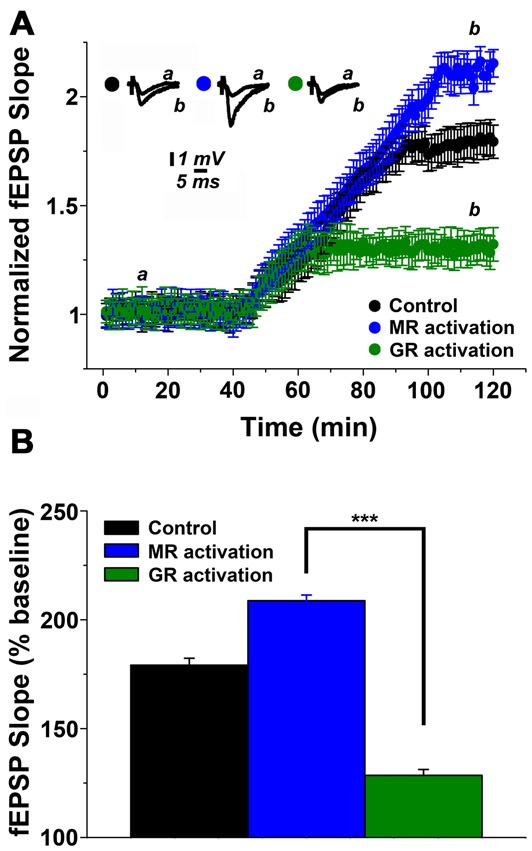
Steroid hormone receptor mediated modulation of mLTP in hippocampal slices. **(A)** MR activation enhances while GR activation depresses mLTP. In these experiments mifepristone (500 nM) and corticosterone (100 nM) were used to activate MR while spironolactone (500 nM) and corticosterone (100 nM) were used to achieve GR-activation. **(B)** Summary graph and combined analysis of experimental data. Further details of the results and the statistical comparisons are described in the text. Statistical analyses were made with one-way ANOVA, followed by *post hoc* Tukey’s comparisons. ***p < 0.0001.

In order to confirm and extend the above findings, which suggest that stress affects hippocampal cholinergic plasticity via corticosteroid receptor activation, we next performed *in vitro* pharmacological experiments in hippocampal slices prepared from non-stressed control animals. In these experiments mifepristone (500 nM) and corticosterone (100 nM) was used to activate MR, or spironolactone (500 nM) and corticosterone (100 nM) to achieve GR activation (Maggio and Segal, 2007). As shown in Figure 3, MR activation resulted in a 1.99 ± 0.091 (at 100 min of recordings) mLTP, while GR stimulation resulted in 1.29 ± 0.075 of muscarinic potentiation (Figure 3A). One-way ANOVA comparing the results in control slices and those obtained in slices where MR and GR were stimulated, revealed significant differences among groups (*F* = 17.2, *p* < 0.0001, Figure 3B). Hence, similar to our *in vivo* pharmacological experiments this set of experiments disclosed the differential effect of MR and GR on mLTP.

### GR Activation Modulates N-methyl-D-aspartate Receptor (NMDAR) Activity

**Figure 4:**
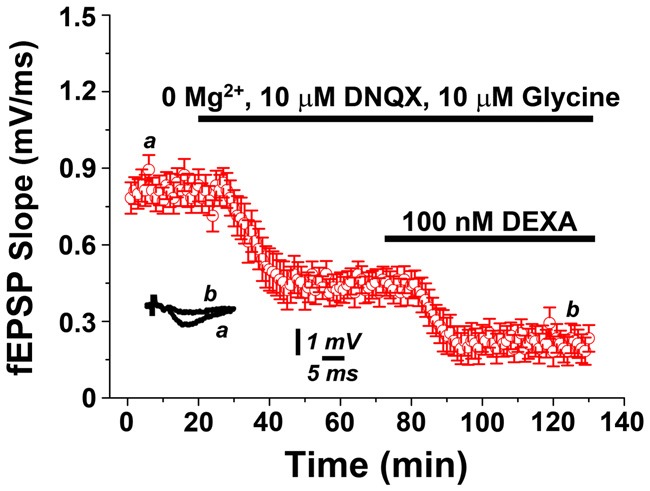
GR activation depresses N-methyl-D-aspartate receptor (NMDAR)-mediated potentials. NMDAR-mediated field potentials recorded from Schaffer collateral-CA1 synapses in presence of 0 μM Mg^2+^, 10 μM DNQX and 10 μM Glycine are depressed upon exposure to the GR agonist dexamethasone (100 nM).

It has been previously shown that mLTP results from the cholinergic activation of N-methyl-D-aspartate receptors (NMDARs), which leads to enhanced synaptic responses following exposure of hippocampal slices to 0.5 μM carbachol (Markram and Segal, 1990; Auerbach and Segal, 1996). Therefore, we predicted that GR-activity depresses mLTP by reducing NMDAR-evoked potentials. In order to test this hypothesis, we recorded evoked NMDAR potentials in presence of 0 μM Mg^2+^, 10 μM DNQX and 10 μM Glycine, as previously described (Maggio and Segal, 2007). Indeed, in this setting, exposure to 100 nM dexamethasone resulted in a depression of NMDAR-mediated potentials (Figure 4). We conclude that GR activation may act in our experimental setting by modulating NMDARs.

### Multiple, Unpredictable Stress Persistently Impairs mLTP

**Figure 5:**
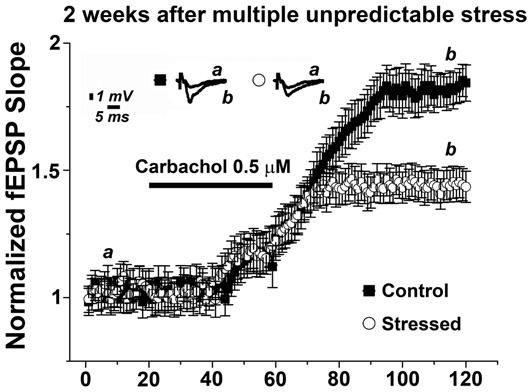
Multiple stressful experiences persistently impair mLTP. mLTP evoked by 0.5 μM carbachol in animals exposed to multiple stressful experiences 2 weeks prior slice preparation is significantly reduced compared to the mLTP evoked in age- and time-matched control, non-stressed animals. Averaged fEPSP are plotted vs. time. Representative traces at indicated times (a, b) are shown on top of each section, *n* = 12 slices for each experiment.

Finally, we wondered whether the effects of multiple, unpredictable stress on mLTP are transient or long-lasting. Thus, we exposed animals to the multiple stressful procedure and performed hippocampal slices 2 weeks later to test for the ability to induce mLTP (Figure 5). Strikingly, in these experiments 0.5 μM carbachol resulted still in a lower magnitude mLTP compared to non-stressed animals (1.45 ± 0.06 and 1.81 ± 0.09, respectively, *p* < 0.001). These results show that the effects of multiple, unpredictable stress are not only seen after a few hours following the last exposure to stress. We conclude that multiple stressful events can have long-lasting effects on hippocampal cholinergic plasticity.

## Discussion

In this study, we addressed the role of stress in modulating mLTP. We found that a single exposure to stress does not influence mLTP, while multiple stressful procedures reduce the magnitude of mLTP. This effect on mLTP is long-lasting, since it can be detected even two weeks following the stressful events. Corticosteroids are involved in mediating the effect of multiple, unpredictable stress on mLTP. Inhibition of GR prevents alterations in mLTP, while GR activity blocks mLTP likely via a reduction in NMDA-R currents. Considering the relevance of ACh-mediated synaptic plasticity under physiological and pathological conditions (Markram and Segal, 1990; Auerbach and Segal, 1996; Hut and Van der Zee, 2011), these results provide new important insight on how chronic unpredictable stress may contribute to the development of cognitive decline and dementia.

It has been previously reported that repeated stress predisposes to the onset of dementia and AD. The mechanisms underlying this phenomenon are not completely understood (Simard et al., 2009; Solas et al., 2010; Millan et al., 2012). The results of the present study reveal that stress directly affects cholinergic plasticity and may thus impair ACh-mediated learning and memory (Hut and Van der Zee, 2011). Alterations of cholinergic transmission are considered a first, crucial step for the onset of Alzheimer’s dementia (Millan et al., 2012). As such stress may contribute to the pathophysiology of the disease from its early stages. If this is indeed the case, stress management should be timely adopted in order to counteract chronic stress and therefore prevent (or at least postpone) the onset of the disease.

The mechanisms through which chronic stress modulates hippocampal cholinergic plasticity warrant further investigation. In this study, we were able to provide first evidence that corticosteroids are involved in stress-mediated alterations of hippocampal mLTP. While MR activation promotes mLTP, GR activation impairs the ability of neurons to express mLTP. Yet, the precise molecular mechanisms through which these differential effects of corticosteroid receptors are mediated are unknown. It is interesting to speculate though that they may resemble effects described in the context of tetanus-induced synaptic plasticity (Joëls and Krugers, 2007; Maggio and Segal, 2012b). Consistent with the results of the present study, it has been previously shown that GR activation impairs LTP by depressing NMDAR potentials (Maggio and Segal, 2007). Conversely, MR activation promotes LTP through the activation of voltage-gated calcium channels (VGCCs; Joëls and Krugers, 2007; Groeneweg et al., 2012; Maggio and Segal, 2012a). Since, it has been reported that muscarinic M1 receptor can enhance synaptic plasticity through VGCCs (Giessel and Sabatini, 2010), it is conceivable that the positive effects of MR on mLTP may involve this pathway. Nevertheless, additional experiments are needed in order to clarify this hypothesis.

Another interesting observation of the present study concerns differences between a single stressful event and multiple, unpredictable stressful procedures. Previous work has demonstrated that exposure to a single stressful event affects tetanic stimulation induced LTP (Maggio and Segal, 2007). Unexpectedly, this is not the case for mLTP. A possible explanation is that mLTP does not only depend on NMDARs. Hence, additional mechanisms may compensate for GR mediated changes in NMDARs under conditions of single stressful events, e.g., changes in VGCCs. Under conditions of chronic stress these compensatory mechanisms may fail or could even be directly or indirectly affected by multiple, unpredictable stress. In order to better understand the effects of stress on cholinergic plasticity it will be important to assess compensatory mechanisms and the ability of neurons to express homeostatic plasticity. It is for example conceivable that different compensatory mechanisms may also act at different points in time following acute or chronic stress. For example, in this study, we performed slices 1 day after the exposure to single stress, while in previous studies we assessed tetanic LTP 1 h upon stress exposure. Hence, in the current conditions, synapses may recover from the stressful experience and as such exhibit a normal NMDAR-dependent mLTP. This theory is supported by our findings in juvenile animals where a normal stimulus-induced LTP could be evoked 1 day after stress in the dorsal hippocampus (Maggio and Segal, 2011). As the case may be, additional work is needed in order to better understand the time-course of alterations in mLTP and putative compensatory mechanisms.

Yet, the effects of multiple stress exposure on mLTP resemble those reported on tetanus-induced LTP. Specifically, we (Maggio and Segal, 2011) and others (Pavlides et al., 2002; Alfarez et al., 2003; Karst and Joëls, 2003; Krugers et al., 2010) have shown that chronic, multiple exposure to stress impairs tetanus-induced LTP. Therefore it is tempting to speculate that multiple stress exposure may reduce the ability of neurons to express NMDAR-dependent synaptic plasticity, regardless of the stimulus used to induce/probe plasticity thus providing a cellular substrate for the development of both hippocampal—dependent cognitive (Sotiropoulos et al., 2011) and emotional (Albrecht et al., 2017) deficits after a prolonged exposure to stressful events.

## Author Contributions

AV and NM: designed the study and wrote the article. ESS, ZIH and NM: performed and analyzed experiments.

## Conflict of Interest Statement

The authors declare that the research was conducted in the absence of any commercial or financial relationships that could be construed as a potential conflict of interest.
